# Associations of the Social Determinants of Health and Lifestyle Components with Dietary Patterns in a Population of Reproductive Age

**DOI:** 10.3390/nu17060950

**Published:** 2025-03-08

**Authors:** Anca-Elena Crăciun, Adriana Rusu, Cornelia Bala, Dana Mihaela Ciobanu, Cristian-Ioan Crăciun, Adriana Fodor, Gabriela Roman, Camelia Vonica, Georgeta Inceu

**Affiliations:** 1 2nd Department, Faculty of Nursing and Health Sciences, “Iuliu Hațieganu” University of Medicine and Pharmacy, 4 Louis Pasteur Street, 400349 Cluj-Napoca, Romania; anca.craciun@umfcluj.ro; 2 Department of Diabetes and Nutrition Diseases, Faculty of Medicine, “Iuliu Hațieganu” University of Medicine and Pharmacy, 2-4 Clinicilor Street, 400006 Cluj-Napoca, Romania; cbala@umfcluj.ro (C.B.); dana.ciobanu@umfcluj.ro (D.M.C.); adriana.fodor@umfcluj.ro (A.F.); groman@umfcluj.ro (G.R.); cami_umf@yahoo.com (C.V.); inceu.victoria@umfcluj.ro (G.I.); 3 Department of Pharmacology, Toxicology and Clinical Pharmacology, Faculty of Medicine, “Iuliu Hațieganu” University of Medicine and Pharmacy, 23 Gheorghe Marinescu Street, 400337 Cluj-Napoca, Romania; doctor.craciun@yahoo.com

**Keywords:** dietary patterns, social determinants, lifestyle, reproductive age, eating jetlag, social jetlag

## Abstract

Background/Objectives: Lifestyle factors, sociodemographic determinants, and dietary patterns play an important role in shaping genitors and fetal health. This study aimed to identify dietary patterns and to investigate the social determinants of health and lifestyle components associated with dietary patterns and body mass index (BMI) in a population of reproductive age. Methods: A cross-sectional online survey was conducted between March 2021 and February 2022. Self-reported data on age, weight, height, social determinants, lifestyle factors, and medical history were collected. Results: A total of 284 participants of reproductive age (≤40 years of age) were included in the analysis. We identified 3 main dietary patterns: (1) the Prudent pattern, associated with a higher probability of eating 3 meals/day, a longer eating jetlag and a higher probability of being a homemaker, unemployed, or a student; (2) the Western pattern, associated with eating after 9 p.m., a longer eating jetlag and negatively associated with the employment status (i.e., of being a homemaker, unemployed, or a student); and (3) the Unhealthy pattern, associated with being a smoker. Furthermore, using multivariate linear regression, we found that BMI was associated with living in rural area and adopting an Unhealthy dietary pattern. For the Unhealthy pattern, the adherence to it was higher in smoking men > smoking women, with a significant interaction between gender and smoking status (*p* < 0.001). Conclusions: These data could be helpful in implementing personalized educational interventions in nutrition and lifestyle changes tailored for risk categories in order to improve health in people of reproductive age.

## 1. Introduction

The nutritional habits of a population represent a cornerstone of public health and play a pivotal role in determining health outcomes, including obesity and related chronic conditions [1]. Understanding dietary patterns and their determinants is critical for informing tailored public health interventions and policies. The recent literature has emphasized the value of examining dietary patterns, rather than isolated food items, as a more comprehensive approach for understanding dietary behaviors and their impact on health [2].

Obesity, a significant global health issue, has multifactorial origins, including lifestyle factors, sociodemographic determinants, and dietary habits, each playing a distinct role in shaping maternal and fetal health. Lifestyle factors, such as physical activity, substance use, and sleep patterns, directly impact the physiological processes of pregnancy [3]. Sociodemographic determinants, including socioeconomic status, education, and access to healthcare, influence the availability of resources and the ability to maintain a healthy pregnancy [4,5]. Dietary habits, encompassing nutritional intake and food security, are critical for supporting fetal development and reducing the risk of complications such as low birth weight or gestational diabetes [6]. Young adults of reproductive age have a critical role in the perpetuation of future generations, but are particularly susceptible to the cumulative effects of poor dietary choices and lifestyle behaviors. Furthermore, social determinants of health, such as educational attainment, employment status, and urban versus rural living, profoundly shaped dietary choices and body mass index (BMI) through a multifaceted interplay of factors. For instance, lower educational attainment may limit awareness of healthy dietary practices, while unemployment can exacerbate food insecurity, leading to reliance on calorie-dense, nutrient-poor foods [7]. Urban living, though associated with better access to diverse food options, can also increase exposure to fast-food outlets, contributing to unhealthy eating habits. Conversely, rural living might restrict access to fresh products, further influencing BMI trajectories and overall health outcomes for both maternal and fetal populations [8].

The general health status of both females and males at the reproductive age plays a key role in fertility and have impact on pregnancy outcome. The most recent data provided by the World Health Organization estimates that 1 in 6 people of reproductive age are affected by infertility [9]. In half of the cases, the cause of infertility is unknown, but unhealthy lifestyle is considered among potential risk factors [10]. Lifestyle factors, such as dietary patterns, physical inactivity, cigarette smoking, alcohol consumption, sleeping habits, and the perception of psychological stress are considered modifiable factors that have a significant impact on health [11].

Romania, a country with 19 million inhabitants, 55.2% living in urban areas, with diverse cultural and socioeconomic landscapes, provides a unique setting for investigating the interplay of dietary patterns, lifestyle factors, and social determinants of health [12,13,14,15]. Although it is estimated that the median age of the population is 43.2 years, below the European Union (EU) mean age of 44.7 years, the life expectancy in Romania was the third lowest in the EU in 2022 [14,15,16]. Despite a growing body of research on diet and health in Romania, studies specifically targeting young adults of reproductive age remain scarce.

To fill this gap, here, we aimed to identify dietary patterns and their predictors (social determinants and lifestyle factors) among young Romanian adults of reproductive age. We also aimed to explore lifestyle components as well as social determinants associated with higher BMI in this population.

## 2. Materials and Methods

### 2.1. Study Design, Participants and Recruitment Process

The study design and protocol were previously described [13]. Briefly, this was a cross-sectional online survey performed in Romania between March 2021 and February 2022. Persons 18 years of age and older were invited to complete the questionnaire via face-to-face meetings, through advertisement on social media and emails. The exclusion criteria used to control for the effect of the cofounding variables on the main study objective [17,18], i.e., social jetlag (SJL), were pregnancy or breastfeeding, jetlag resulting from more than two time zones flights within two weeks before study inclusion, working on shifts, and refusal to participate.

Before questionnaire completion, all participants were informed about data collection and processing and their rights according to the applicable data privacy regulation. The Ethics Committees of the Iuliu Hatieganu University of Medicine and Pharmacy Cluj-Napoca, Romania approved this study and all related materials (No 15/18.01.2021).

### 2.2. Data Collection, Lifestyle Factors and Social Determinants of Health

As previously described [13], all data were collected using a questionnaire distributed online, self-declared by the participants, and included age, weight and height, social determinants, lifestyle factors, and medical history (previous diagnosis of ischemic heart disease, stroke, hypertension, diabetes, and other chronic diseases). BMI was calculated as weight (kg)/height (m)^2^ and a BMI ≥ 30 kg/m^2^ denoted obesity [19].

Lifestyle factors assessed included eating and sleep behavior, dietary intake, smoking status (current smoker/nonsmoker), and self-perceived stress.

Eating behavior was assessed by assessing the number of meals/day, the largest meal of the day (breakfast, lunch or dinner), breakfast consumption (daily, most days, rarely), waking up during the night to eat (night eating) (yes/no), eating while watching TV/playing on the computer/reading (yes/no) [20], and meal timing during weekdays (working) and weekends (rest days). Based on data on meal timing, we computed eating jetlag as the absolute difference between the midpoint of eating time during weekends and weekdays [21].

A modified version of a previously validated food-frequency questionnaire [22] was used to assess food and beverage intake in the last 12 months. This questionnaire evaluates the intake frequency of standard portions of 59 food and beverage items (bread, cereals, pasta, eggs, meat, fish, milk and dairy products, sweets, snacks, cooked food, legumes, vegetables, fruits, fried and fast food, and type of fat used for cooking). With 2 exceptions, the frequency of consumption ranged between “never” scored as 1 to “6 or more times per day”, scored as 9. For the items “fried food consumption” and “fast-food consumption”, scores ranged between 1 (less than once/week) and 4 (daily). For this analysis, based on the previous literature [23], the 59 food and beverage items were aggregated into 30 mutually exclusive food groups with similar nutrient content. A score was calculated for each food group by adding the scores of individual food items as previously described elsewhere [23]. Missing data were not imputed.

Sleep behavior in the previous month was assessed using questions from the Munich Chronotype Questionnaire (MCQT) [24] on bedtime, sleep-onset latency, and wake-up time, for weekdays and weekends. Based on this information, we calculated the sleep-onset time (bedtime plus sleep-onset latency), SJL corrected for sleep duration (SJLsc) [25], and weighted sleep duration [26].

Social determinants of health assessed based on the Healthy People conceptual framework [27] were the place of residence (urban or rural), the highest education level attained (secondary school, professional education, high school, university), and the employment status (homemaker, unemployed, unable to work, student, retired, employed).

Data collected were exported in an excel file and imported in the statistical analysis software. All filled-in questionnaires were reviewed for fulfilling the inclusion/exclusion criteria, complete information on sleep habits (the main study objective), and possible duplicates (based on timestamp and data provided). All participants which presented exclusion criteria or provided incomplete or unrealistic data (4–7 h or 480 min for sleep latency) on sleep habits were excluded along with possible duplicates.

### 2.3. Statistical Analysis

Statistical analysis was performed using SPSS version 26.0 (Armonk, NY, USA: IBM Corp). The quantitative variables are presented as mean ± SD or as median (quartile 1; quartile 3). The categorical and ordinal variables are presented as the number of observations and frequency.

As this is a post hoc analysis of data collected for a survey aiming to assess the prevalence of SJLsc in the Romanian population, no specific sample-size calculation for the current analysis was performed. The sample was calculated for the main study objective [13].

The assessment of dietary patterns specific to the young Romanian sample of reproductive age enrolled was performed using an a posteriori approach by principal component analysis with Varimax rotation, which allowed for dimension reduction. Based on the eigenvalue > 1 and the interpretability [28], we retained 3 principal factors (dietary patterns) labeled based on food groups with a high factor loading. Based on previous literature, we chose factor loading with an absolute value ≥ 0.4 [29] as a cut-off, indicating a good correlation of the food group with the dietary pattern (principal factor). These food groups were considered to contribute significantly to the dietary pattern and were thus retained and used to calculate factor scores using the regression procedure with Bartlett correction. Factor scores could display negative or positive values, indicating a lower or higher adherence to the respective dietary pattern.

The association of lifestyle factors and social determinants of health with dietary patterns and BMI was investigated using the multivariate linear regression analysis adjusted for age. Subsequently, we tested whether gender modifies the associations observed by assessing the interaction using general linear models.

For all analyses, a *p*-value < 0.05 was considered statistically significant.

## 3. Results

**Figure 1:**
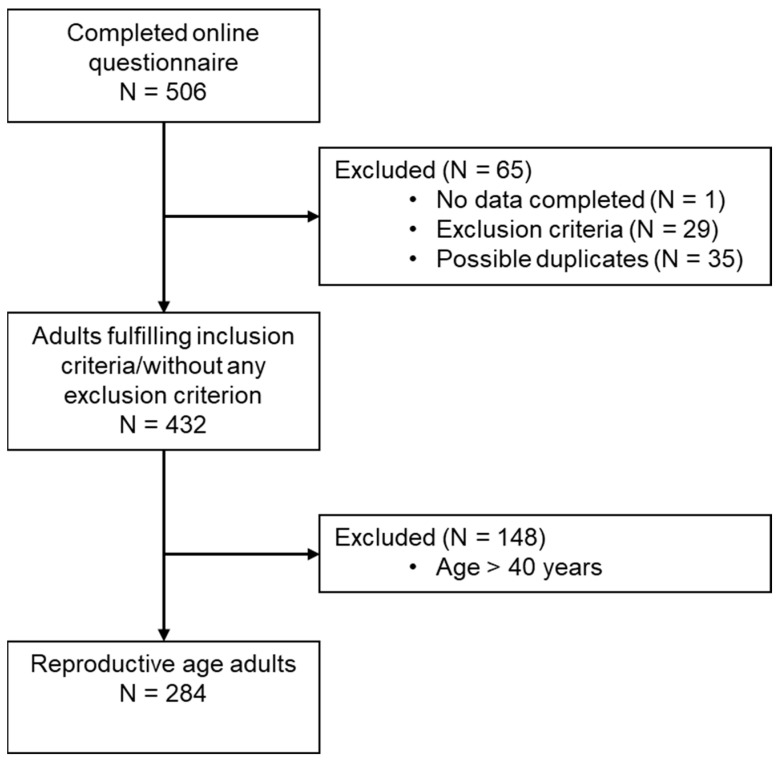
Study participants’ disposition.

Participant disposition is presented in Figure 1. As previously described [13], of the 506 persons who completed the online questionnaire, 1 had no data completed, 29 were excluded due to exclusion criteria (23 worked in shifts, 1 person traveled between time zones two weeks prior to survey completion, 9 did not provide sleep-onset latency data or provided unrealistic data, and 5 were pregnant or lactating) and 35 possible duplicates were removed (based on timestamp and information provided). Thus, data from 432 adults 18 to 71 years of age from all regions of Romania, with complete sleep data, who fulfilled the inclusion criteria and were without any self-declared exclusion criteria, were retained for any analysis. For the analysis presented here, we included 284 participants considered to be of reproductive age (≤40 years of age). Most of the sample were women (71.5%), from urban areas, with an average age of 30.0 years. Most of them were employed (76.4%) and had higher education (75.7%). Regarding lifestyle habits, 20.4% declared themselves current smokers and over half reported feeling stressed. The average time spent sedentary per day was 8.0 h, while weighted sleep duration was 7.7 h/night. SJLsc and eating jetlag duration were 43.2 and 50.6 min, respectively.

Most of the participants declared eating three meals/day and having breakfast every day or most days. The most important meal of the day was lunch for most of the participants, and only 5% woke up during night to eat. As medical history, the most frequently found pathology was obesity (10.2%), with cardiovascular diseases being rarely reported due to young age (Table 1).

Using principal component analysis with orthogonal Varimax rotation, and based on the eigenvalue > 1 and the interpretability, three factors were retained (three dietary patterns), explaining 36.2% of the diet variance. The Kaiser–Meyer–Olkin test had a value of 0.775, indicating that the sample was adequate for factor analysis, while Bartlett’s test of sphericity had a *p*-value < 0.001, showing that the correlation matrix is not an identity matrix and confirming that the variables are related and thus suitable for the factor analysis. Factor loadings for individual food groups are presented in Table 2.

The dietary pattern identified by Factor 1 was labeled the Prudent pattern and explained 18.3% of the diet variance. This pattern was characterized by a high intake of vegetables, legumes, fruits, whole wheat pasta, whole rice, breakfast cereals, cheese, milk and milk products, eggs, nuts, and dark chocolate. The dietary pattern identified by Factor 2 explained 10.9% of the diet variance, labeled the Western pattern and characterized by a high intake of processed meat, potatoes, fried potatoes, white wheat bread, biscuits and snacks, milk chocolate/other sweets, soft drinks, fried food, and fast food. The dietary pattern identified by Factor 3 explained 7.0% of the diet variance, labeled the Unhealthy pattern and characterized by a high intake of processed meat, fish, white wheat pasta and white rice, and alcoholic beverages (wine, beer, and spirits).

Among lifestyle factors and social determinants of health analyzed, the Prudent dietary pattern was associated with a higher probability of eating three meals/day (β = 0.248, *p* < 0.001), a longer eating jetlag (β = 0.191, *p* = 0.007), and a higher probability of being a homemaker, unemployed, or a student (β = −0.173, *p* = 0.031). The Western pattern was associated with eating after 21:00 (β = 0.197, *p* = 0.005), a longer eating jetlag (β = 0.147, *p* = 0.042), and negatively associated with employment status (i.e., of being a homemaker, unemployed, or a student; β = −0.166, *p* = 0.043). The Unhealthy pattern was associated with being a smoker (β = 0.283, *p* < 0.001) (Table 3).

We further analyzed whether gender modifies the association of lifestyle factors and social determinants of health with dietary patterns identified. For the Prudent pattern, we observed a higher adherence to it in men and women eating daily three meals/day (mean factor score 0.324 in men vs. 0.288 in women) and a significant interaction of gender with the probability of eating three meals/day (*p* = 0.005) and eating jetlag duration (*p* = 0.005). No interaction was observed with the employment status (*p* = 0.168). For the Western dietary pattern, we observed a higher adherence to it in women eating daily after 21:00 and in men eating most of the time after 21:00 and a significant interaction of gender with eating after 21:00 (*p* = 0.023). However, no gender interaction with the professional situation (*p* = 0.078) and eating jetlag (*p* = 0.236) was observed. For the Unhealthy pattern, the adherence to it was higher in smoking men > nonsmoking men > smoking women > nonsmoking women, with a significant interaction between gender and smoking status (*p* < 0.001).

Using multivariate linear regression, we also analyzed the association of BMI with selected lifestyle and sociodemographic characteristics. BMI was associated with living in rural area (β = 0.132; *p* = 0.040), and adopting an Unhealthy dietary pattern (β = 0.256; *p* < 0.001; Table 4).

We also tested whether there was any interaction between gender and the variables significantly associated with BMI. We observed a higher BMI in women from rural areas and men living in urban areas and a significant interaction between gender and living area (*p* < 0.001) and the adoption of an Unhealthy dietary pattern (*p* = 0.016).

## 4. Discussion

Using data from an online survey we identified three dietary patterns among young adults of reproductive age: a Prudent one, a Western one, and an Unhealthy one, as well as social determinants and lifestyle components associated with each of them. The Prudent pattern was characterized by a high intake of vegetables, legumes, fruits, whole cereals, breakfast cereals, milk and milk products, eggs, nuts, and dark chocolate. This pattern was adopted with a higher probability by homemakers, the unemployed, or students, with a longer eating jetlag and a higher probability of eating three meals/day (with a higher adherence to this pattern in men than in women eating three meals/day). The Western one was characterized by a high intake of processed meat, potatoes, white wheat bread, sweets, soft drinks, fried food, and fast food. This pattern was adopted with a higher probability by those eating after 21:00 (with a higher adherence to this pattern in women than in men eating after 21:00), a longer eating jetlag, homemakers, the unemployed, or students. The Unhealthy dietary pattern was characterized by a high intake of processed meat, fish, white wheat pasta and white rice, and alcoholic beverages, and more probably followed by those who declared themselves smokers, with a higher adherence to this pattern in smoking men than in smoking women.

The association of dietary patterns with social determinants of health has recently emerged as a research subject in correlation with health outcomes. For instance, a study in Mexico City identified that higher education and income levels are associated with healthier diets rich in fruits and vegetables, while lower socioeconomic status correlates with increased consumption of processed foods [30]. In urban Burkina Faso, a cross-sectional study identified three primary dietary patterns: “modern”, “traditional”, and “intermediate”. Notably, higher adherence to the “modern” pattern, characterized by processed foods, was associated with younger age, higher education, and formal employment, while the “traditional” pattern, rich in local grains and vegetables, was more common among older individuals with lower socioeconomic status [31]. In Brazil, research involving young adults revealed two main dietary patterns: “healthy” encompassing fruits, vegetables, and whole grains, and “unhealthy” including sweets and processed foods. Higher income and education levels were positively associated with the “healthy” pattern, whereas lower socioeconomic status correlated with the “unhealthy” pattern [32]. Similarly, a study in Fars Province, Iran, examined the socioeconomic determinants of nutritional behaviors, highlighting that higher income and education levels were linked to healthier dietary choices, whereas lower socioeconomic status was associated with increased consumption of unhealthy foods [33]. We did not identify any association of educational status with the dietary patterns in our sample, probably due to the imbalance in the educational attainment observed; a higher proportion of our respondents graduated university. However, we observed a higher adherence to a Western pattern in respondents who were less likely to be employed and had limited economic resources to procure healthy foods. A major concern is that the Western pattern and the Unhealthy one were also associated with other unhealthy lifestyle factors which have been shown to be associated with non-communicable chronic diseases, such as eating after 21:00 and being a smoker.

Due to an increase in infertility cases with unknown causes, an increase in the investigation of unhealthy lifestyle as a key contributing factor has been observed in the past few years. A recent systematic review and meta-analysis which screened 15,396 studies in order to evaluate if dietary patterns impact fertility outcomes found 10 different diet patterns associated with improved natural conception and assisted reproductive technology outcomes that can be broadly grouped into three categories: the Mediterranean pattern, the Healthy pattern, or the Unhealthy pattern. For the Mediterranean diet, they found that higher adherence was associated with better pregnancy rates and pregnancy outcomes [34]. The total fertility rate (TFR) is a parameter that estimates the average number of children that a woman would have if she would live through all the years of reproductive age according to age-specific fertility rates. Globally, TFR decreased from 5 in 1950 to 2.3 in present and is projected to decrease to 2.1 by 2050 [35]. In Romania, in 2021, the TFR was estimated to be 1.8, slightly higher than EU’s TFR of 1.5 [14]. Dietary patterns play a role in fertility and TFR, with evidence that a Mediterranean diet has beneficial effects, while a Western-type diet has a negative impact [36,37]. In our study, the Prudent diet was characterized by a high intake of vegetables, legumes, fruits, whole wheat pasta, whole rice, breakfast cereals, cheese, milk and milk products, eggs, nuts, and dark chocolate, displaying large similarities with the Mediterranean diet. In a large prospective cohort following 17,544 women without a history of infertility for eight years, adhering to a dietary pattern named the “fertility diet” was found to have a 66% lower risk of infertility related to ovulatory disorders, irrespective of age, parity, or BMI. This dietary pattern was rich in plant protein from vegetable sources and high-fat dairy, monounsaturated fats rather than trans fats, complex carbohydrates, multivitamins, and iron supplements [38].

We also found that 1 out of 10 participants had obesity, defined as a BMI ≥ 30 kg/m^2^. In another study performed in the Romanian population, the prevalence of obesity reported in 2014 in 18–39-year-old age group was 9.9% (in the context of 21.3% prevalence of obesity in the whole adult population) [22]. A higher BMI may result from limited physical activity in urban areas with high fast-food availability, or from food insecurity in lower socioeconomic groups leading to reliance on calorie-dense foods [37]. These interrelated factors underscore the need to contextualize BMI within the broader framework of health equity and lifestyle behaviors, as they significantly shape maternal and fetal health outcomes. Obesity is one of the most important causes of infertility in both males and females. In a meta-analysis of 21 studies and a sample of 13,077 men, the risk for oligozoospermia or azoospermia increased by 28% in obese men and doubled in extremely obese men [39]. In females, a meta-analysis of 18 studies indicated that overweight/obesity is associated with 44% increased risk of subfecundity and 60% increased risk of infertility [40].

In our study, a higher BMI was significantly associated with female gender living in rural area and male gender living in urban areas and adopting an Unhealthy dietary pattern. In a systematic scoping review, Tully CA et al. found that the preconception diet has some impact on male fertility. Reducing processed meats, total fat and sugary drinks may improve male fertility and healthy diets (including fish, fatty acids, carbohydrates, dairy, and reduced content of processed meat) can improve sperm health [41]. In our study, male gender was associated with both of the less-healthy diets, the Western and Unhealthy patterns. A recent published study aiming to evaluate dietary and lifestyle quality among the Romanian population in the post COVID-19 pandemic period showed low adherence to a healthy diet (20.7%) or a healthy lifestyle (12.28%), especially among young participants [42]. In our study, the Unhealthy pattern was also associated with other cardiovascular risk factors, such as smoking, with a potential cumulative negative effect on the fertility rate and a lower educational attainment. The same observations were made a French nationwide cohort study involving 998 fathers, where smoking and low educational level was positively associated with “Processed products” patterns, while high educational levels was related to a “Balanced” pattern. Maternal and paternal dietary patterns have many dissimilarities and need to be approached by different interventions [43].

Interestingly, in our study, the eating jetlag (50.6 min) and SJLsc (43.2 min) were not associated with BMI in this young population. In a study involving 1106 young adults (aged 18–25 years), Zerón-Rugerio MF et al. investigated the effect of eating jetlag on BMI and found a significant positive association, but BMI significantly increased after a 3.5 h threshold of eating jetlag [21]. In a large study, with 65,000 participants from central European countries, one third of the people had a SJL more than 2 h, and 69% had a SJL of at least 1 h. In the same study, the presence of SJL was a strong predictor for being overweight [44]. In our study, the average SJLsc was relatively small (below 1 h), but was associated with an Unhealthy dietary pattern, which was associated with a higher BMI. Furthermore, a recent published study showed that an increasing trajectory or chronic SJL (≥2 h of difference in sleep midpoint between weekends and weekdays) after the age of 11 years is associated with obesity at age of 23 years, meaning that the prevention of obesity should be started in adolescence [45].

Although the prevalence of smoking has decreased in the last thirty years (by 27.5% in men and by 37.7% in women), the total number of smokers increased, due to the global rise in the population. It is estimated that in 2019, 1.14 billion people were current smokers, with 155 million being young people, aged 15–24 years (equivalent to 20.1% of young males and 5.0% of young females) [46]. Also, one in five participants in our study declared themselves to be active smokers. Beyond oncogenic and cardiovascular risk, the cigarettes contain thousands of components that can impact all stages of reproduction [47]. Augood C et al. observed in their study that the risk of infertility in women increases by 60% in smokers versus non-smokers and there was a 34% reduction in the number of pregnancies/number of IVF-treated cycles in smokers versus non-smokers [48].

The World Health Organization (WHO) states that people with insufficient physical activity have a 20 to 30% increased risk of death and estimates that 31% of adults and 80% of adolescents do not meet the levels of physical activity recommended by the guidelines [49]. It was previously found that prolonged sitting time, more than 7 h per day, leads to an increase in all-cause mortality by 5% [50]. In our study, the mean duration of time spent in sedentary activities was 8 h. Furthermore, sedentary behavior at fertile age is associated with a double risk of infertility in men and triple risk of infertility in women [51]. The global target declared by WHO is to reduce time spent in sedentarism by 15% in adults and adolescents, by 2030 [52].

The limitations of the study include the online collection of self-reported data from participants with the skills to use electronic devices and with internet access and an interest in health topics. Those enrolled were mainly employed adults with higher education, notably women, and those with digital access barriers, lower socioeconomic status, lower educational attainment, or less interest in the health topics were more likely to not be approached. Therefore, our online survey, like all online surveys, may have a limited representativity for the general population of reproductive age. As previously described, this imbalance in the selection is characteristic to the online surveys and may be due to both a selection bias and an interest bias [51]. Also, the potential recall bias is typical for surveys collecting retrospective self-reported data [53]. Recall ability decreases over time, and thus some people may fail to report past events, such as the amount and frequency of some food rarely consumed. Also, some people may erroneously report consumption of foods, though this never happened during the survey reference period; this retrieval bias is due to the combination of past experiences from a previous period with the reference period [54]. Incorrect answers may be due to the social desirability bias as well [55]. However, our anonymous survey administration is a technique proposed to mitigate this type of bias and limit the tendency of respondents to provide answers that may be viewed as favorable by those analyzing the responses [56]. Another limitation is the cross-sectional design of the study which does not allow for a statement of causal relationship between social determinants of health, dietary patterns and BMI. We also acknowledge that additional information on energy intake, macro-, and micronutrients collected through the interviews on the dietary intake for the past 3–5 days would provide important information for the reproductive potential of the population. The collection of data on the dietary intake used for this analysis was performed online as part of a larger study, and thus a food-frequency questionnaire was considered a more appropriate strategy for this approach, which allows for a limited analysis of these parameters. However, it may represent a starting point for the design of future prospective studies investigating the causality and assessing the dietary intake in detail.

Despite the study limitations, the policy and clinical implications of these findings are profound. Targeted interventions, such as subsidizing healthy foods in lower-income communities and improving access to grocery stores in food deserts, have been suggested as effective strategies. Community-based nutrition programs tailored to cultural and economic contexts can also improve dietary behaviors. A study that evaluated a culturally tailored nutrition education program for low-income African American families found significant improvements in participants’ dietary habits, including increased fruit and vegetable consumption [57]. Clinically, healthcare providers should integrate social determinants of health into dietary counseling, offering realistic and accessible nutritional advice for at-risk populations. The Centers for Disease Control and Prevention (CDC) emphasizes the importance of addressing social determinants of health to improve health outcomes. The CDC proposes a framework to address social determinants of health with six pillars: surveillance, data collection and dissemination; the evaluation and elaboration of strategies to reduce disparities and promote equity in health; collaboration to improve health outcomes; community engagement and infrastructure for implementation and policy support [58]. These findings underscore the necessity of multi-sectoral approaches to reduce dietary disparities and improve public health outcomes.

## 5. Conclusions

In conclusion, in the present study we identified three dietary patterns with different associations with other lifestyle factors and social determinants of health. The Prudent pattern was adopted with a higher probability by homemakers, the unemployed, or students, with a longer eating jetlag and a higher probability of eating three meals/day (with a higher adherence to this pattern in men than in women eating three meals/day). The Western pattern was adopted with a higher probability by those eating after 21:00 (with a higher adherence to this pattern in women than in men eating after 21:00), a longer eating jetlag, homemakers, the unemployed, or students. The Unhealthy dietary pattern was more probably followed by those who declared themselves smokers, with a higher adherence to this pattern in smoking men than in smoking women. Furthermore, in our study, a higher BMI was significantly associated with female gender living in rural area and male gender living in urban areas, and adopting a Unhealthy dietary pattern. Overall, this study provides up-to-date information about lifestyle and associations of social determinants of health with dietary patterns in a young population. These data could be helpful in implementing personalized educational measures in nutrition and lifestyle changes tailored for risk categories in order to improve health in young people of reproductive age. By exploring these relationships, this study contributes to the understanding of dietary behaviors and their health implications, which is crucial for designing effective public health strategies.

## Figures and Tables

**Table 1:** Selected lifestyle components, social determinants of health, and medical history in men and women of reproductive age.

	Total N = 284
Age, years	30.0 ± 6.3
Women, n (%)	203 (71.5%)
Living in urban area, n (%)	229 (80.6%)
Employment status, n (%) Housewife Unemployed Unable to work Student Employed	10 (3.5%) 3 (1.1%) 0 53 (18.7%) 217 (76.4%)
Education, n (%) Secondary school Professional education High school University	1 (0.4%) 4 (1.4%) 64 (22.5%) 215 (75.7%)
Time spent sedentary per day, h	8.0 ± 3.2
Current smoker, n (%)	58 (20.4%)
Feeling stressed, n (%)	170 (59.9%)
Weighted sleep duration, h	7.7 ± 1.0
SJL, min	43.2 ± 41.0
Eating jetlag, min	50.6 ± 43.1
Eating 3 meals/day, n (%) Daily Most days	84 (29.6%) 128 (45.1%)
Eating breakfast, n (%) Daily Most days	123 (43.3%) 66 (23.2%)
The most important meal of the day, n (%) Breakfast Lunch Dinner	28 (9.9%) 194 (68.3%) 62 (21.8%)
Eating after 21:00, n (%) Daily Most days	9 (3.2%) 48 (16.9%)
Waking up during the night for eating, n (%)	14 (4.9%)
Eating while watching TV, reading or in front of computer, n (%)	156 (54.9%)
BMI, kg/m^2^	23.8 ± 4.4
Medical history, n (%) Obesity Ischemic heart disease Stroke High blood pressure Diabetes Other diseases	29 (10.2%) 3 (1.1%) 3 (1.1%) 15 (5.3%) 3 (1.1%) 79 (27.8%)

n/N (%) = number (percentage) of participants in each category; BMI = body mass index; SJL = social jetlag; h = hour; min = minute.

**Table 2:** Rotated component matrix—food items loading on each identified factor.

	Prudent Pattern (Factor 1)	Western Pattern (Factor 2)	Unhealthy Pattern (Factor 3)
Red meat Poultry Processed meat Fish Potatoes Fried potatoes Vegetables Legumes Fruits White wheat pasta and white rice Whole wheat pasta and whole rice Breakfast cereals Polenta White bread Whole wheat bread Full-fat cheese Low-fat cheese Milk and milk products High-fat food Soy milk Biscuits and snacks Milk chocolate and sweets Dark chocolate Nuts Eggs Wine Beer Spirits Soft drinks Fried foods Fast food	0.107 0.313 −0.028 0.342 0.254 −0.043 0.670 0.592 0.623 0.354 0.434 0.460 0.292 0.151 0.366 0.537 0.573 0.617 0.298 0.318 0.372 0.394 0.437 0.536 0.473 0.057 −0.198 −0.093 −0.092 −0.111 −0.214	0.293 0.017 0.502 0.037 0.530 0.728 −0.224 −0.133 −0.085 0.354 −0.043 −0.011 0.307 0.506 0.134 0.167 0.100 0.044 0.396 0.050 0.579 0.473 0.100 0.153 0.056 −0.002 0.168 0.112 0.563 0.691 0.598	0.249 0.212 0.505 0.494 0.013 0.098 0.321 0.316 0.169 0.417 0.379 −0.037 0.096 −0.121 −0.084 0.069 0.015 −0.108 0.366 −0.081 −0.021 0.043 −0.098 0.110 0.106 0.701 0.627 0.754 0.329 0.091 0.059

**Table 3:** Association of selected lifestyle factors and social determinants of health with dietary patterns in adults of reproductive health.

Variables Included in the Multivariate Regression Models	Standardized β Coefficient	*p*-Value
**Prudent pattern** Eating 3 meals/day Waking up during nights for eating Eating after 21:00 Eating while watching TV Time spent sedentary per day, h Current smoker, n (%) Feeling stressed, n (%) Weighted sleep duration, h SJLsc, min Eating jetlag, min Living area Employment status Education	0.248 −0.026 −0.011 0.025 −0.111 −0.068 0.001 0.074 0.001 0.191 −0.043 −0.173 0.003	<0.001 0.692 0.871 0.717 0.098 0.314 0.987 0.273 0.994 0.007 0.518 0.031 0.971
**Western pattern** Eating 3 meals/day Waking up during nights for eating Eating after 21:00 Eating while watching TV Time spent sedentary per day, h Current smoker, n (%) Feeling stressed, n (%) Weighted sleep duration, h SJLsc, min Eating jetlag, min Living area Employment status Education	−0.051 −0.004 0.197 0.109 −0.049 0.064 −0.081 0.089 −0.112 0.147 0.072 −0.166 0.034	0.450 0.950 0.005 0.126 0.472 0.350 0.242 0.199 0.114 0.042 0.289 0.043 0.688
**Unhealthy pattern** Eating 3 meals/day Waking up during nights for eating Eating after 21:00 Eating while watching TV Time spent sedentary per day, h Current smoker, n (%) Feeling stressed, n (%) Weighted sleep duration, h SJLsc, min Eating jetlag, min Living area Employment status Education	0.090 −0.048 0.103 −0.004 0.005 0.283 0.071 0.081 0.134 −0.051 −0.069 0.099 −0.111	0.175 0.469 0.125 0.960 0.945 <0.001 0.291 0.229 0.052 0.465 0.297 0.212 0.183

All multivariate regression models were adjusted for age. Variables coding: eating three meals/day: 1 = rarely/2 = most of the days/3 = daily; waking up during nights for eating: no = 0/1 = yes; eating after 21:00: 1 = rarely/2 = most days/3 = daily; eating while watching TV: no = 0/1 = yes; gender: women = 1/men = 2; education: primary school or gymnasium = 1 professional education = 2//secondary school = 3/university = 4; living area: urban = 1/rural = 2; employment status: homemakers = 1/unemployed = 2/not capable of working (disease/handicap) = 3/student = 4/retired = 5/employed or freelancer = 6; current smoking: no = 0/yes = 1; feeling stressed: no = 0/yes = 1; age, time spent sedentary per day, weighted sleep duration, and social and eating jetlag were included as continuous variables.

**Table 4:** Association of lifestyle factors and social determinants of health with BMI in adults of reproductive health.

Variables Included in the Multivariate Regression Model	Standardized β Coefficient	*p*-Value
Eating 3 meals/day	−0.093	0.157
Waking up during night for eating	−0.032	0.611
Eating after 21:00	0.009	0.891
Eating while watching TV	0.093	0.165
Time spent being sedentary per day, h	0.017	0.791
Current smoker, n (%)	−0.009	0.892
Feeling stressed, n (%)	−0.014	0.824
Weighted sleep duration, h	−0.038	0.557
SJLsc, min	−0.091	0.173
Eating jetlag, min	0.041	0.553
Living area	0.132	0.040
Employment status	0.001	0.988
Education	0.027	0.738
Prudent pattern	−0.086	0.196
Western pattern	0.060	0.354
Unhealthy pattern	0.256	<0.001

Multivariate regression models were adjusted for age. Variables coding: eating 3 meals/day: 1 = rarely/2 = most of the days/3 = daily; waking up during nights for eating: no = 0/1 = yes; eating after 21:00: 1 = rarely/2 = most of the days/3 = daily; eating while watching TV: no = 0/1 = yes; gender: women = 1/men = 2; education: primary school or gymnasium = 1/professional education = 2/secondary school = 3/university = 4; living area: urban = 1/rural = 2; employment status: homemakers = 1/unemployed = 2/not capable of working (disease/handicap) = 3/student = 4/retired = 5/employed or freelancer = 6; current smoking: no = 0/yes = 1; feeling stressed: no = 0/yes = 1; age, time spent sedentary per day, weighted sleep duration, and social and eating jetlag were included as continuous variables.

## Data Availability

The data that support the findings of this study are available from the corresponding author, upon reasonable request.

## References

[1] Wang P., Song M., Eliassen A.H., Wang M., Fung T.T., Clinton S.K., Rimm E.B., Hu F.B., Willett W.C., Tabung F.K. (2023). Optimal dietary patterns for prevention of chronic disease. Nat. Med..

[2] Shang X., Liu J., Zhu Z., Zhang X., Huang Y., Liu S., Wang W., Zhang X., Tang S., Hu Y. (2023). Healthy dietary patterns and the risk of individual chronic diseases in community-dwelling adults. Nat. Commun..

[3] Whitaker K.M., Zhang D., Kline C.E., Catov J., Barone Gibbs B. (2021). Associations of Sleep With Sedentary Behavior and Physical Activity Patterns Across Pregnancy Trimesters. Womens Health Issues.

[4] Flor-Alemany M., Nestares T., Jiménez N.M., Baena-García L., Aparicio V.A. (2022). Associations between Sociodemographic Factors, Lifestyle Behaviors, Pregnancy-Related Determinants, and Mediterranean Diet Adherence among Pregnant Women: The GESTAFIT Project. Nutrients.

[5] Simpson S.E., Malek A.M., Wen C.C., Neelon B., Wilson D.A., Mateus J., Pearce J., Chundru K.J., Korte J.E., Florez H. (2024). Trends in Gestational Weight Gain and Prepregnancy Obesity in South Carolina, 2015-2021. Prev. Chronic. Dis..

[6] Soltani H., Smith D., Olander E. (2017). Weight, Lifestyle, and Health during Pregnancy and Beyond. J. Pregnancy.

[7] Azizi Fard N., Mejova Y., Schifanella R. (2021). On the interplay between educational attainment and nutrition: A spatially-aware perspective. EPJ Data Sci..

[8] Jiao L. (2024). Social Determinants of Health, Diet, and Health Outcome. Nutrients.

[9] World Health Organization Infertility—Key Facts. 22 May 2024. https://www.who.int/news-room/fact-sheets/detail/infertility.

[10] Cannarella R., Crafa A., Curto R., Condorelli R.A., La Vignera S., Calogero A.E. (2024). Obesity and male fertility disorders. Mol. Aspects. Med..

[11] Sharma R., Biedenharn K.R., Fedor J.M., Agarwal A. (2013). Lifestyle factors and reproductive health: Taking control of your fertility. Reprod. Biol. Endocrinol..

[12] Roman G., Bala C., Craciun A., Craciun C.I., Rusu A. (2016). Eating Patterns, Physical Activity And Their Association With Demographic Factors In The Population Included In The Obesity Study In Romania (ORO STUDY). Acta Endocrinol..

[13] Ciobanu D., Porojan M., Bala C., Zah A.M., Oroian I., Roman G., Rusu A. (2024). Lifestyle factors, dietary patterns, and social determinants of social and eating jetlag: A cross-sectional survey. Chronobiol. Int..

[14] OECD State of Health in the EU. https://health.ec.europa.eu/system/files/2023-12/2023_chp_ro_english.pdf.

[15] Worldometer Romania Population. https://www.worldometers.info/world-population/romania-population/.

[16] Eurostat EU’s Median Age Increased to 44.1 Years in 2021. https://ec.europa.eu/eurostat/web/products-eurostat-news/-/ddn-20220228-1.

[17] Baron K.G., Reid K.J. (2014). Circadian misalignment and health. Int. Rev. Psychiatry.

[18] Won C.H. (2015). Sleeping for Two: The Great Paradox of Sleep in Pregnancy. J. Clin. Sleep Med..

[19] World Health Organization (2000). Obesity: Preventing and managing the global epidemic. Report of a WHO consultation. World Health Organ. Tech. Rep. Ser..

[20] Roman G., Rusu A., Graur M., Creteanu G., Morosanu M., Radulian G., Amorin P., Timar R., Pircalaboiu L., Bala C. (2019). Dietary patterns and their association with obesity: A cross-sectional study. Acta Endocrinol..

[21] Zerón-Rugerio M.F., Hernáez Á., Porras-Loaiza A.P., Cambras T., Izquierdo-Pulido M. (2019). Eating Jet Lag: A Marker of the Variability in Meal Timing and Its Association with Body Mass Index. Nutrients.

[22] Roman G., Bala C., Creteanu G., Graur M., Morosanu M., Amorin P., Pîrcalaboiu L., Radulian G., Timar R., Achimas Cadariu A. (2019). Obesity and health-related lifestyle factors in the general population in Romania: A cross sectional study. Acta Endocrinol..

[23] Atkins J.L., Whincup P.H., Morris R.W., Lennon L.T., Papacosta O., Wannamethee S.G. (2016). Dietary patterns and the risk of CVD and all-cause mortality in older British men. Br. J. Nutr..

[24] Roenneberg T., Wirz-Justice A., Merrow M. (2003). Life between clocks: Daily temporal patterns of human chronotypes. J. Biol. Rhythms..

[25] Jankowski K.S. (2017). Social jet lag: Sleep-corrected formula. Chronobiol. Int..

[26] Reutrakul S., Hood M.M., Crowley S.J., Morgan M.K., Teodori M., Knutson K.L., Van Cauter E. (2013). Chronotype is independently associated with glycemic control in type 2 diabetes. Diab. Care.

[27] Centers of Disease Control and Prevention Healthy People 2030 Framework. https://odphp.health.gov/healthypeople/about/healthy-people-2030-framework.

[28] Fransen H.P., May A.M., Stricker M.D., Boer J.M., Hennig C., Rosseel Y., Ocké M.C., Peeters P.H., Beulens J.W. (2014). A posteriori dietary patterns: How many patterns to retain?. J. Nutr..

[29] Cutillo L., Ranganathan S., Gribskov M., Nakai K., Schönbach C. (2019). Parametric and multivariate methods. Encyclopedia of Bioinformatics and Computational Biology.

[30] Martínez-Vargas L., Vermandere H., Bautista-Arredondo S., Colchero M.A. (2022). The role of social determinants on unhealthy eating habits in an urban area in Mexico: A qualitative study in low-income mothers with a young child at home. Appetite.

[31] Weil K., Coulibaly I., Fuelbert H., Herrmann A., Millogo R.M., Danquah I. (2023). Dietary patterns and their socioeconomic factors of adherence among adults in urban Burkina Faso: A cross-sectional study. J. Health Popul. Nutr..

[32] Arruda S.P.M., da Silva A.A., Kac G., Goldani M.Z., Bettiol H., Barbieri M.A. (2014). Socioeconomic and demographic factors are associated with dietary patterns in a cohort of young Brazilian adults. BMC Public Health.

[33] Foroozanfar Z., Moghadami M., Mohsenpour M.A., Houshiarrad A., Farmani A., Akbarpoor M.A., Shenavar R. (2022). Socioeconomic determinants of nutritional behaviors of households in Fars Province, Iran, 2018. Front. Nutr..

[34] Winter H.G., Rolnik D.L., Mol B.W.J., Torkel S., Alesi S., Mousa A., Habibi N., Silva T.R., Oi Cheung T., Thien Tay C. (2023). Can Dietary Patterns Impact Fertility Outcomes? A Systematic Review and Meta-Analysis. Nutrients.

[35] Anderson R.A., Hickey M. (2023). Reproduction in a changing world. Fertil. Steril..

[36] Toledo E., Lopez-del Burgo C., Ruiz-Zambrana A., Donazar M., Navarro-Blasco I., Martínez-González M.A., de Irala J. (2011). Dietary patterns and difficulty conceiving: A nested case-control study. Fertil. Steril..

[37] Cristodoro M., Zambella E., Fietta I., Inversetti A., Di Simone N. (2024). Dietary Patterns and Fertility. Biology.

[38] Chavarro J.E., Rich-Edwards J.W., Rosner B.A., Willett W.C. (2007). Diet and lifestyle in the prevention of ovulatory disorder infertility. Obstet. Gynecol..

[39] Sermondade N., Faure C., Fezeu L., Shayeb A.G., Bonde J.P., Jensen T.K., Van Wely M., Cao J., Martini A.C., Eskandar M. (2013). BMI in relation to sperm count: An updated systematic review and collaborative meta-analysis. Hum. Reprod. Update.

[40] Zhou J., Zhang Y., Teng Y., Dou L., Chen H., Tao F., Huang K. (2024). Association between preconception body mass index and fertility in adult female: A systematic review and meta-analysis. Obes. Rev..

[41] Tully C.A., Alesi S., McPherson N.O., Sharkey D.J., Teong X.T., Tay C.T., Silva T.R., Puglisi C., Barsby J.P., Moran L.J. (2024). Assessing the influence of preconception diet on male fertility: A systematic scoping review. Hum. Reprod. Update.

[42] Mititelu M., Popovici V., Neacșu S.M., Musuc A.M., Busnatu Ș.S., Oprea E., Boroghină S.C., Mihai A., Streba C.T., Lupuliasa D. (2024). Assessment of Dietary and Lifestyle Quality among the Romanian Population in the Post-Pandemic Period. Healthcare.

[43] de Lauzon-Guillain B., Krinitzki S., Lioret S., Charles M.A. (2022). Paternal Diet before Conception and Its Social Determinants in the Elfe Cohort. Nutrients.

[44] Roenneberg T., Allebrandt K.V., Merrow M., Vetter C. (2012). Social jetlag and obesity. Curr. Biol..

[45] Chang C.S., Chang L.Y., Wu C.C., Chang H.Y. (2024). Associations between social jetlag trajectories and body mass index among young adults. Sleep.

[46] GBD 2019 Tobacco Collaborators (2021). Spatial, temporal, and demographic patterns in prevalence of smoking tobacco use and attributable disease burden in 204 countries and territories, 1990–2019: A systematic analysis from the Global Burden of Disease Study 2019. Lancet.

[47] Dechanet C., Anahory T., Mathieu Daude J.C., Quantin X., Reyftmann L., Hamamah S., Hedon B., Dechaud H. (2011). Effects of cigarette smoking on reproduction. Hum. Reprod. Update.

[48] Augood C., Duckitt K., Templeton A.A. (1998). Smoking and female infertility: A systematic review and meta-analysis. Hum. Reprod..

[49] World Health Organization Physical Activity—Key Facts. 26 June 2024. https://www.who.int/news-room/fact-sheets/detail/physical-activity.

[50] Goyal J., Rakhra G. (2024). Sedentarism and Chronic Health Problems. Korean. J. Fam. Med..

[51] Andrade C. (2020). The limitations of online surveys. Indian J. Psychol. Med..

[52] Foucaut A.M., Faure C., Julia C., Czernichow S., Levy R., Dupont C., ALIFERT collaborative group (2019). Sedentary behavior, physical inactivity and body composition in relation to idiopathic infertility among men and women. PLoS ONE.

[53] Moreno-Serra R., Anaya-Montes M., León-Giraldo S., Bernal O. (2022). Addressing recall bias in (post-)conflict data collection and analysis: Lessons from a large-scale health survey in Colombia. Confl. Health.

[54] Bradburn N.M., Huttenlocher J., Hedges L., Schwarz N., Sudman S. (1994). Telescoping and temporal memory. Autobiographical Memory and the Validity of Retrospective Reports.

[55] Krumpal I. (2013). Determinants of social desirability bias in sensitive surveys: A literature review. Qual. Quant..

[56] Nederhof A.J. (1985). Methods of coping with social desirability bias: A review. Eur. J. Soc. Psychol..

[57] Winham D.M. (2009). Culturally tailored foods and CVD prevention. Am. J. Lifestyle Med..

[58] Centers of Disease Control and Prevention Social Determinants of Health (SDOH) at CDC. https://www.cdc.gov/about/priorities/social-determinants-of-health-at-cdc.html.

